# Retro-Appendicular Internal Hernia: A Rare Cause of Small Bowel Obstruction

**DOI:** 10.70352/scrj.cr.25-0396

**Published:** 2025-10-09

**Authors:** Jade Heinicke, Jean-Marc Heinicke

**Affiliations:** Department of Surgery, Hopital de la Tour, Geneva, Switzerland

**Keywords:** internal hernia, retro-appendicular hernia, small bowel obstruction, laparoscopic surgery

## Abstract

**INTRODUCTION:**

Internal hernias are rare causes of small bowel obstruction and are often difficult to diagnose preoperatively. Retro-appendicular internal hernias are exceedingly rare, with very few reports in the literature. This case is noteworthy for involving a patient with no prior history of abdominal surgery, highlighting an unusual presentation of a retro-appendicular internal hernia.

**CASE PRESENTATION:**

A man in his mid-60s, with no history of abdominal surgery, presented to the emergency department with acute right lower quadrant abdominal pain. Physical examination revealed localized peritonism, and laboratory results were unremarkable. CT showed signs of small bowel distension with segmental hypoperfusion of the bowel loop in the right lower quadrant. Given the clinical presentation of an acute abdomen, the patient underwent exploratory laparoscopy, which revealed a herniation of the small intestine into the retro-appendicular space. The herniated bowel appeared ischemic but was successfully reduced without further need for resection. The patient had an uneventful postoperative recovery.

**CONCLUSIONS:**

This case underscores the importance of considering internal hernias in the differential diagnosis of acute abdomen, even in patients without prior abdominal surgery. Awareness of rare anatomical variants such as retro-appendicular hernias is critical, as early surgical exploration can prevent bowel necrosis and reduce morbidity. This report contributes to the limited research literature on retro-appendicular internal hernias and emphasizes the value of maintaining a high index of suspicion in atypical presentations.

## Abbreviation

RLQ
: right lower quadrant

## INTRODUCTION

Internal hernias are an uncommon cause of small bowel obstruction, and even more rare are hernias into the retro-appendicular and retrocaecal space.^1,2)^ Cases are most likely seen following previous abdominal surgery. Diagnosis is based on clinical signs of intestinal obstruction, peritonism, and radiological assessment of the bowel, which can be challenging. The risk of delay in treatment is small bowel necrosis. We have found the pathology described in a few case reports, specifically retrocaecal hernias given their small occurrence.^3,4)^ The retro-appendicular hernia belongs to the category of retrocaecal hernias, but no other case report was found describing this subtype specifically.

## CASE PRESENTATION

**Fig. 1:**
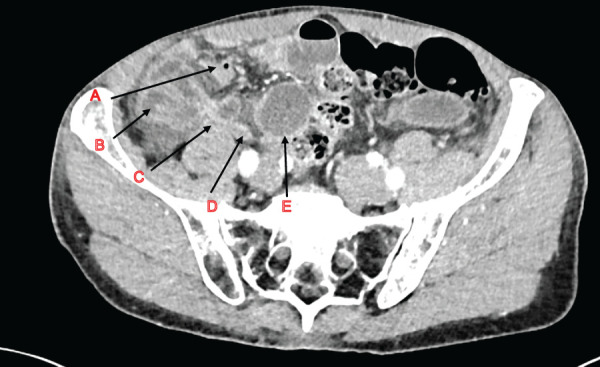
Preoperative CT scan showing (arrows): (**A**) gas-filled appendix, (**B**) incarcerated bowel loop, (**C**) hernial orifice, (**D**) flattened distal bowel loop, and (**E**) dilated proximal bowel loop.

Our patient, aged in the mid-60s, was known only for a prophylactic thoracic aortic replacement 12 years ago when he presented to the emergency department with acute abdominal pain. The patient had been awakened by an acute onset of pain in the right lower quadrant (RLQ). He had no fever, no nausea, and no alteration of stool. He had never had any abdominal surgery. Clinical examination showed peritonism in the RLQ. His lab workup showed no inflammatory markers and no abnormal liver panel. CT examination showed no sign of appendicitis, but described distension of the small intestine in the RLQ with segmental hypoperfusion of the bowel loop in the RLQ (**Fig. 1**).

**Fig. 2:**
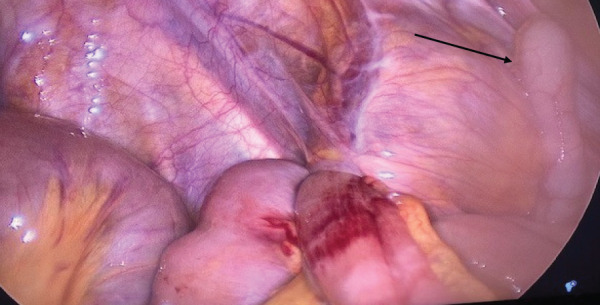
Incarcerated bowel loop in the retro-appendicular space. Non-inflammatory appendix (arrow).

**Fig. 3:**
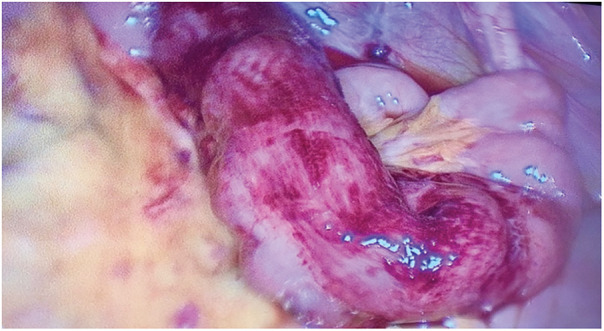
Reduced loop showing signs of acute ischemia with serosal suffusions due to strangulation.

**Fig. 4:**
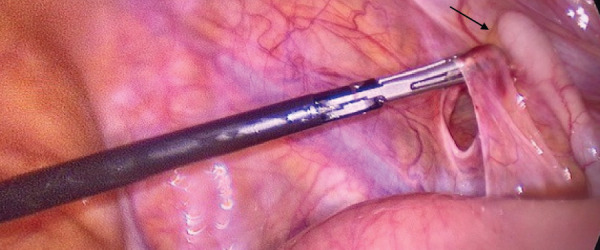
Retro-appendicular defect with the 2-cm hole through which the herniation had occurred (arrow showing appendix).

**Fig. 5:**
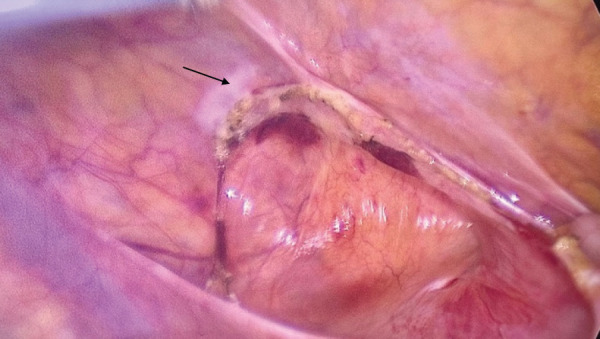
Resected retroperitoneal defect (arrow) with marks of dissection into the retro-appendicular recess to avoid recurrence.

**Fig. 6:**
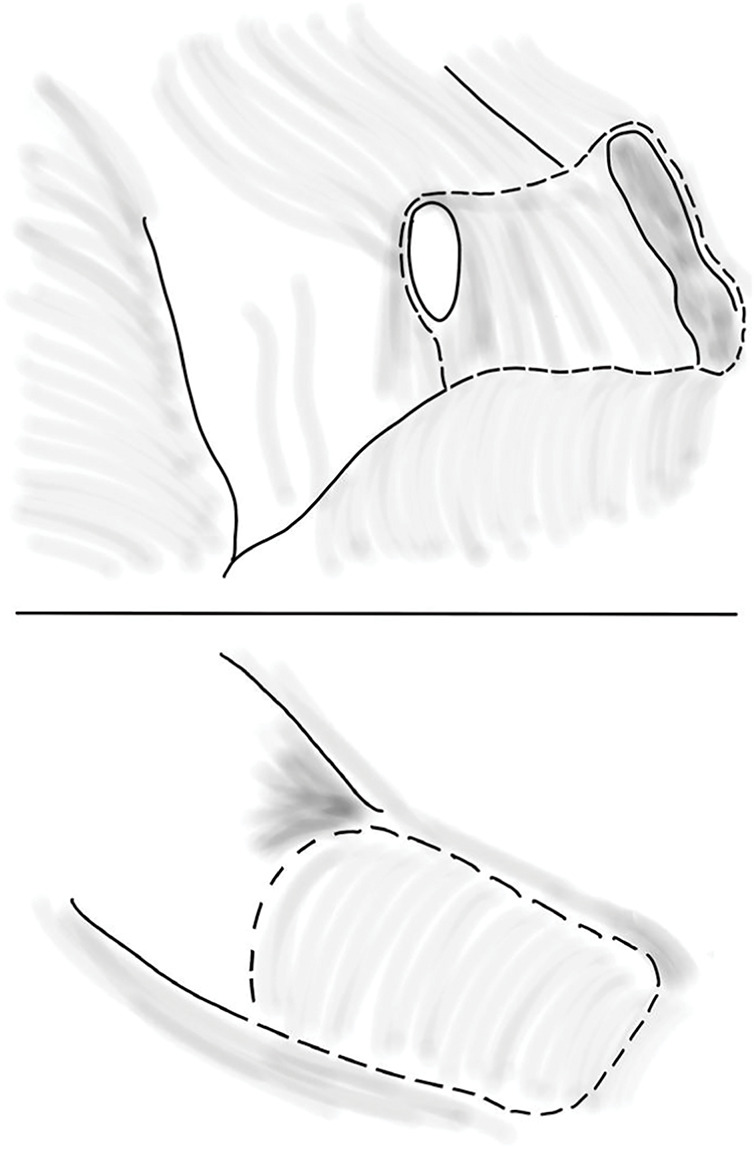
Schematic illustration of the resection site (- - -), including the peritoneal defect and the appendix.

The patient was taken to the operating room for exploratory laparoscopy, given clear signs of an acute abdomen. Peroperatively, an internal hernia of the small intestine into the retro-appendicular space was found (**Fig. 2**). Through gentle traction, the incarcerated loop was freed. The small intestine showed signs of acute ischemia, but recovered perioperatively, so that no resection was necessary (**Fig. 3**). The opening that had caused the herniation was a 2-cm small peritoneal defect (**Fig. 4**). The peritoneal opening and the appendix were resected en bloc: The appendicular peritoneal adhesion containing the opening into the retroperitoneal space was resected using scissors, until it merged with the retro-appendicular recess (**Fig. 5**), thus preventing recurrence (**Fig. 6**). A standard laparoscopic appendectomy was performed. The patient presented no postoperative complications.

## DISCUSSION

Internal hernias are rare, especially in patients with no prior history of abdominal surgery. Risk factors for internal trans-mesenteric hernias include previous abdominal surgery, specifically gastric bypass surgery or liver transplantation. Some cases of spontaneous internal hernias may be due to defects in the peritoneal ligaments fixating the caecum and the appendix during embryogenesis.^1)^ Clinical and radiological diagnosis can be challenging, and knowledge of this entity is primal in the treatment plan. A high index of suspicion paired with proper collaboration between radiologists and surgeons is key to ensuring swift management.^2)^ Indeed, clinical signs of peritonism associated with a CT scan showing dilated bowel loops and hypoperfusion in the RLQ should bring to mind small bowel obstruction through herniation, especially in the retro-caecal and retro-appendicular space, thus necessitating emergent operative care.^5)^ Unfortunately, no transition point, closed loop, or mesenteric swirl was visible on our imaging; however, these signs are clear indicators of bowel obstruction and—if occurring in the RLQ—should add to clinical suspicion of retro-appendicular hernias. This illustrates the diagnostic difficulty of this entity, and active research of direct (transition point) or indirect signs (mesenteric swirl, dilated loops) of bowel obstruction needs to be evaluated in collaboration with the radiologist. Differential diagnoses, especially in patients with prior surgery, include obstruction due to adhesions, retro-caecal hernia, inguinal hernia, and femoral hernia. Herniation can occur through any peritoneal defect as small as 2 cm, as shown in our case report. This case is noteworthy for involving a patient with no prior history of abdominal surgery, highlighting a spontaneous and unusual presentation of retro-appendicular internal hernia and underscoring the importance of a high clinical suspicion.

## CONCLUSIONS

This case emphasizes the importance of considering internal hernias in the differential diagnosis of acute abdomen, even in patients without prior abdominal surgery. Awareness of rare anatomical variants such as retro-appendicular hernias is critical, as early surgical exploration can prevent bowel necrosis and reduce morbidity. This report contributes to the limited body of literature on retro-appendicular internal hernias and emphasizes the value of maintaining a high index of suspicion in atypical presentations.

## DECLARATIONS

### Availability of data and materials

The datasets and figures supporting the conclusions of this article are included within the article.
